# Correction: Neurogenic decisions require a cell cycle independent function of the CDC25B phosphatase

**DOI:** 10.7554/eLife.63032

**Published:** 2020-09-17

**Authors:** Frédéric Bonnet, Angie Molina, Melanie Roussat, Manon Azais, Sophie Bel-Vialar, Jacques Gautrais, Fabienne Pituello, Eric Agius

Bonnet F, Molina A, Roussat M, Azais M, Bel-Vialar S, Gautrais J, Pituello F, Agius E. 2018. Neurogenic decisions require a cell cycle independent function of the CDC25B phosphatase. *eLife* **7**:e32937. doi: 10.7554/eLife.32937.

Published 3, July 2018

After publication, we identified typographical errors in Figures 2B and 7A, where the y axis is incorrectly marked, and in the legends Figures 2B, 7A and 7H. We also accidentally moved the horizontal dashed line in Figure 1E, when the figure was rearranged after revisions.

**Original Figure 1E:**

The horizontal dashed line is above 50 on the y-axis

**Corrected Figure 1E:**

The horizontal dashed line is at the level of 50 on the y-axis as described in the legend

**Original Figures 2B and 7A:**

y-axis in Figures 2B and 7A: GFP^+^PH3^+^EdU^+^/GFP^+^EdU^+^ (%)

**Corrected Figures 2B and 7A:**

y-axis in Figures 2B and 7A: GFP^+^PH3^+^EdU^+^/GFP^+^PH3^+^ (%)

**Original legend of Figures 2B and 7A:**

**2B and 7A**: Curves representing the percentage of electroporated GFP^+^EdU^+^PH3^+^ over the total GFP^+^EdU^+ ^cells with increasing EdU exposure times

**Corrected legend of Figures 2B and 7A:**

**2B and 7A**: Curves representing the percentage of electroporated GFP^+^EdU^+^PH3^+^ over the total GFP^+^PH3^+ ^cells with increasing EdU exposure times

**Original 7E legend:**

**H**: Box plots (5/95 percentile) comparing the percentage of Sox2+ or HuC/D+ cells within the electroporated population in the control or CDC25BΔPΔCDK gain-of-function experiments, in the dorsal spinal cord at HH17.

**Corrected 7H legend:**

**H**: Box plots (5/95 percentile) comparing the percentage of Sox2+ or HuC/D+ cells within the electroporated population in the control or CDC25BΔPΔCDK gain-of-function experiments, in the dorsal spinal cord at HH22.

The article has been corrected accordingly.

